# multiVIB: A unified probabilistic contrastive learning framework for atlas-scale integration of single-cell multi-omics data

**DOI:** 10.1101/2025.11.29.691308

**Published:** 2025-12-01

**Authors:** Yang Xu, Stephen J. Fleming, Brice Wang, Erin G. Schoenbeck, Mehrtash Babadi, Bing-Xing Huo

**Affiliations:** 1 Data Sciences Platform, Broad Institute of MIT and Harvard, Cambridge, MA

## Abstract

Comprehensive brain cell atlases are essential for understanding neural functions and enabling translational insights. As single-cell technologies proliferate across experimental platforms, species, and modalities, these atlases must scale accordingly, calling for data integration framework that aligns heterogeneous datasets without erasing biologically meaningful variations. Existing tools typically address narrow integration settings, forcing researchers to assemble *ad hoc* workflows that may generate artifacts. Here, we introduce multiVIB, a unified probabilistic contrastive learning framework that handles diverse integration scenarios. We show that multiVIB achieves state-of-the-art performance while mitigating spurious alignments. Applied to atlas-scale datasets from the BRAIN Initiative, multiVIB demonstrates robust and scalable integration, including integration of diverse data modalities and reliable preservation of species-specific variations in cross-species integration. These capabilities position multiVIB as a scalable, biologically faithful foundation for constructing next-generation brain cell atlases with the growing landscape of single-cell data.

## 1 Introduction

The molecular profile of individual cells underpins the complex neural behaviors, cellular interactions, and regulatory circuits that collectively define the mammalian brain[1]. To decode this logic, the BRAIN Initiative established the Cell Census Network (BICCN) and the succeeding Cell Atlas Network (BICAN) to build comprehensive atlases of the mammalian brains at single cell level with molecular resolution. While landmark studies have produced high-quality reference atlases for the mouse[2] and primate (Johansen *et al*., co-submitted) brains using standardized pipelines within single centers, the broader consortium has generated and shared a far more diverse landscape of single-cell datasets, potentially capturing additional biological variations, awaiting to be incorporated into these central resources[3, 4]. To realize this vision of an inclusive and evolving brain atlas, computational frameworks that can integrate heterogeneous single-cell data and preserve the biological faithfulness are critical. Furthermore, the ultimate goal of constructing these atlases is not only to elucidate brain mechanism, but also to realize their translational value. This requires rigorous comparison between model organisms with the human brain[5], which again necessitates integration tools capable of aligning data across species while strictly preserving the biological specificity that distinguishes them.

A common strategy for integrating heterogeneous single-cell multi-omics datasets is to adapt batch-correction methods, originally developed for RNA-seq data, by treating differences across studies, species, or modalities as covariates, and attempting to correct them as if they were technical batch effects[6–12]. While effective in some integration tasks, extensive benchmarking has shown that this approach frequently collapses distinct cell states and suppresses genuine biological differences[13–17]. Specifically, engineering shared features under the batch-correction-like strategy is feasible but not always straightforward for cross-modal integration[18, 19]. Joint-profiling technologies[20–23] now offer a biologically grounded solution where multiple assays were directly probed from individual cells. These jointly-profiled multi-omics datasets supply paired cells as natural anchors for data integration[24–27]. However, these jointly-profiled datasets cover a limited subset of cell populations and constitute only a small fraction of all single-cell data generated to date. Over-reliance on these partial anchors can produce spurious correspondences cross modalities, where unpaired cell types are incorrectly aligned up together or modality-specific regulatory programs are collapsed into an artificial consensus[28]. Moreover, in cross-species integration, data alignment is typically achieved by focusing on conserved homologous genes[14, 29], which may inadvertently eliminating species-specific biology. Some new approaches improved cross-species alignment by drawing on external biological priors such as curated cell-type annotations or pretrained molecular language models[30, 31]. However, dependency on these scarce external resources, whose development is not scalable, could limit broader application of the tools. As large consortia like BICAN expand toward tens of millions of cells across diverse modalities and species, the risk of generating harmonized yet biologically incorrect embeddings jeopardizes the scientific value of next-generation cell atlases.

To address this challenge, we developed multiVIB, a biologically grounded and technically versatile framework for integrating single-cell datasets across studies, species, and molecular modalities. Built on the variational information bottleneck (VIB) principle[32] and probabilistic contrastive learning[33, 34], multiVIB learns latent representations that reflect shared biology while preserving species-, modality-, and study–specific variation. We demonstrate the advantages of multiVIB through two atlas-scale case studies: first, by integrating multi-omic datasets of the mouse primary motor cortex from multiple studies into a coherent, scalable atlas framework; and second, by recovering the cross-species consensus taxonomy of the primate basal ganglia while preserving species-resolved regulatory programs. These results show that the probabilistic design of multiVIB effectively accommodates integration over diverse scenarios and successfully preserves biologically meaningful variation amid technical differences.

## 2 Results

### 2.1 A biologically grounded probabilistic framework for robust single-cell integration

**Figure 1:**
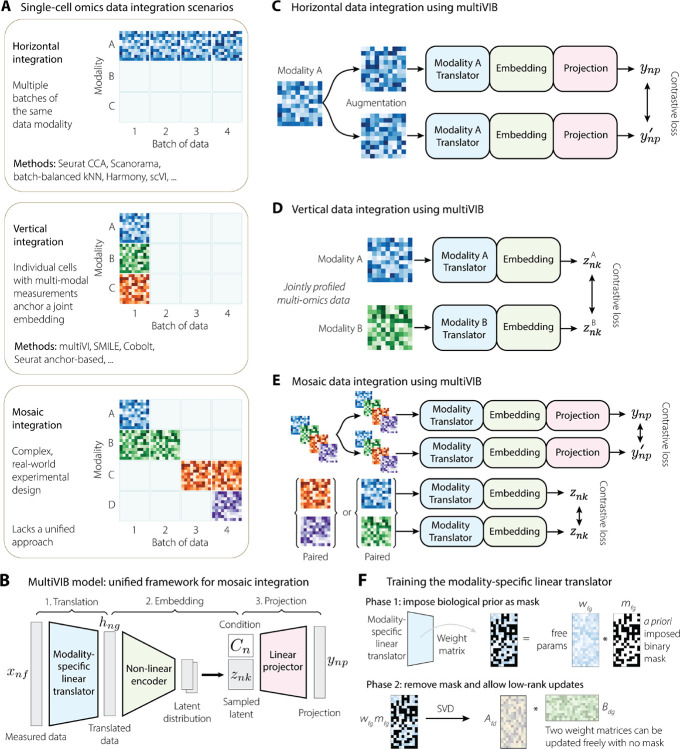
A comprehensive framework for single-cell multi-omics data integration. (A) Three different data integration scenarios are presented. Horizontal integration is the case where there are multiple batches of the same data modality or cross-modal integration through shared features. Vertical integration is the case where individual cells are jointly profiled, collecting several data modalities per cell. Each of these cases have several computational tools available to perform data integration. Mosaic integration is the more complex but common case where there are multiple batches of data, and it may be that different data modalities are collected in different experiments. (B) The components of the multiVIB model. The trainable backbone consists of 3 parts: a modality-specific translator, an encoder, and a projector. Input data $x_{nf}$ is first translated into a gene-centered data $h_{ng}$. Encoder then projects the translated data $h_{ng}$ into latent representation $z_{nk}$. $z_{nk}$ is concatenated with batch, species, or modality covariates C, and projector embeds the concatenated output into a slightly higher dimension space $y_{np}$. After training, $z_{nk}$ becomes the integrated latent representation of cell n. (C) Horizontal integration is accomplished using data augmentation paired with a contrastive loss on projected embeddings $y_{np}$, where the projector is conditioned on each cell’s batch label $C_{n}$. (D) Vertical integration is accomplished using modality-specific translators paired with a contrastive loss on the latent embeddings $z_{nk}$. Training puts positive examples $z_{nk}^{\text{A}}$ and $z_{nk}^{\text{B}}$, which represent the same cell in modalities A and B, close together. (E) Mosaic integration is accomplished using the same framework, with an appropriate, data-dependent mix of the horizontal and vertical strategies. (F) The modality-specific linear translator has two training phases in horizontal integration. The first phase imposes a biologically-informed prior as a mask. The second phase relaxes this constraint. Using the phase 1 weight matrix as a starting point, the weight matrix is decomposed into the low-rank matrices $A_{fd}$ and $B_{dg}$, which are then updated freely.

Single-cell multi-omics data integration strategies can be organized into two principles[35](Fig. 1A): horizontal integration, which aligns datasets through shared genomic features, and vertical integration, which uses jointly-profiled multi-omics cells as direct biological anchors across modalities. Although widely used, neither strategy alone is sufficient for the mosaic structure of real-world scenarios where feature overlap and assay pairing are both partial and inconsistent across studies (Fig. 1A). In these cases, researchers have to select tools and assemble bespoke workflows that can be easily prone to artifacts like spurious alignment. Spurious alignment arises when algorithms over-correct technical differences by collapsing distinct biological features or when disjointed pipelines are applied to complex mosaic datasets for which they were not designed. Note that a third approach, diagonal integration, which attempts to infer correspondences without explicit anchors[35], was excluded from our framework because it is particularly susceptible to spurious matches and is generally unsuitable for atlas-scale tasks[28].

multiVIB was designed as a unified probabilistic framework that operates reliably across horizontal, vertical, and mosaic configurations and produces biologically grounded embeddings. Rather than building separate methods for each integration scenario, multiVIB boasts a single generalizable model backbone, supported by two key principles: (1) uncertainty-aware latent representations, derived through the variational information bottleneck (VIB), which prevent overconfident or forced alignments; and (2) probabilistic contrastive learning (PCL), which reinforces reproducible correspondences across datasets.

At the core of multiVIB is a shared variational encoder that projects all cells, regardless of modality or species, into a common latent space (Fig. 1B). Each dataset, based on its distinct input feature space, would first go through a modality-specific translator that maps heterogeneous measurements—such as chromatin accessibility, methylation, or gene expression—into a gene-centered intermediate space. This gene-centered axis serves as a biologically interpretable scaffold because gene expression represents a common downstream readout across molecular layers and is well represented across datasets. The shared encoder then approximates a stochastic embedding for the translated data to capture biological uncertainty, while a lightweight projector incorporates batch, species, and modality covariates to prevent technical factors from shaping the latent space.

With this backbone fixed, multiVIB adapts to different integration scenarios by altering only the training strategy, not the architecture (Fig. 1C–E). For *horizontal integration* (no jointly-profiled cells; Fig. 1C), datasets are anchored through shared features, and multiVIB aligns cells by enforcing consistency across shared genomic signals while ensuring that technical covariates do not drive the alignment. For *vertical integration* (jointly-profiled multi-modal cells; Fig. 1D), jointly-profiled multi-omics data covers individual cells with multiple modality views. These cells serve as direct biological anchors, allowing multiVIB to learn cross-modality correspondence without relying on engineered feature mappings. *Mosaic integration* (Fig. 1E) is achieved by combining horizontal and vertical steps tailored to the pattern of modality overlap (See Methods 4.1 and ?? for further details).

The novel design of multiVIB, involving VIB and PCL, provides principled safeguards against failure of representation learning that commonly drives spurious alignment. The VIB imposes stochastic and uncertainty-aware embeddings that prevent overconfident alignment, while the contrastive objective reinforces reproducible similarity across datasets. These properties position multiVIB not simply as another option among existing tools, but fundamentally as a more reliable integration backbone. We next show how these design choices lead to more superior and robust integration outcome in different scenarios.

### 2.2 multiVIB mitigates spurious alignment in single-cell integration

Spurious alignment, where biologically distinct cell states are incorrectly mapped to the same latent location, is a pervasive challenge in single-cell data integration. Such error can propagate through downstream analyses and even into the step of constructing reference atlases. To avoid problem of spurious alignment in horizontal integration, we designed two forms of modality-specific translator. The first form is a masked linear translation from input-specific feature space to gene-centered immediate space. The mask is constructed based on biological prior. Take the translation of chromatin accessibility peak data to a gene-centered output as an example, mask informs the model if a chromatin opening region contributes to transcription of a relevant gene. Doing so can prevent the model from exploiting feature correlations solely to fit within-dataset variation. However, adding such mask in training also eliminates recovery of additional within-dataset variations. Therefore, we introduce the second form in the way of lower-rank matrix decomposition. Once an initial biologically consistent alignment was achieved through training translator in the first form, we continue training the model with the second form, which gives the model more freedom to recover additional within-modality variation without breaking the biological coherence (Fig. 1F).

**Figure 2:**
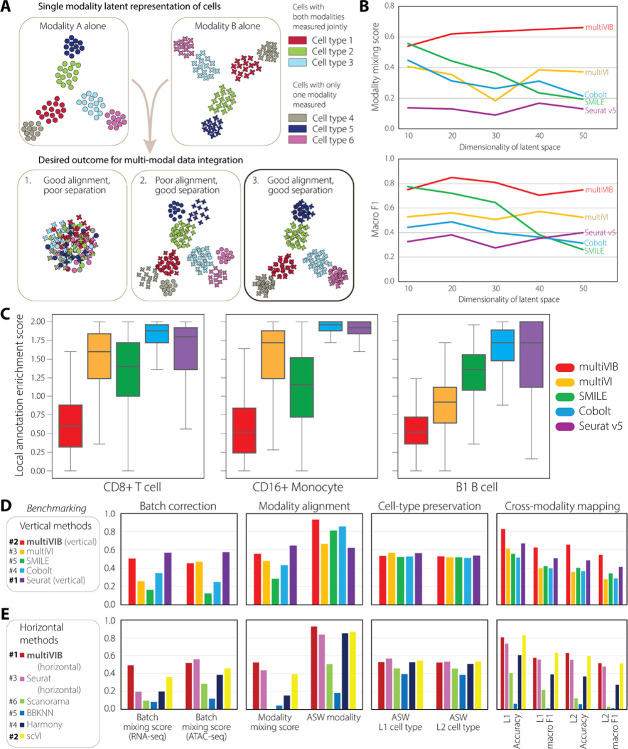
Benchmarking data integration using mutiVIB and existing methods. (A) Conceptual illustration of “spurious alignment” in vertical integration. The thought experiment is that we have a jointly-profiled dataset for modalities A and B. In cell types 1–3, jointly-profiled data are used during data integration, but for cell types 4–6, the model does not know these are jointly-profiled measurements. The joint nature of data collection in cell types 4–6 is withheld and used as ground truth for validation of integration outcome. Data integration can result in various outcomes for cell types 4–6, whose labels are not used during training. Schematic cluster plots show three potential outcomes. “Spurious alignment” would include outcomes 1 and 2, which both are not ideal results. (B) Evaluation of cross-modality alignment in three blinded cell types, similar to the setup in (A), using real 10X Multiome PBMC data. Modality mixing score quantifies the degree of alignment between modalities A and B on the blinded cell types, while label transfer (macro F1) assesses the biological correctness of that alignment. Both metrics are plotted against latent dimensionality to illustrate how embedding dimension also affects integration performance. (C) Local Annotation Enrichment Score (LAES) for the three blinded cell types. LAES quantifies the modality composition of each cell’s nearest neighborhood; lower values indicate better local mixing between two modalities and fewer modality-driven clusters. (D) Benchmarking of 5 vertical integration methods plotting metrics in 4 categories. (E) Similar benchmarking of 6 horizontal methods. L1: coarse cell type annotations; L2: granular cell type annotations.

In vertical integration, which relies on limited jointly-profiled cells, hidden mismatches or underrepresented cell populations can lead to incorrect cross-modal correspondences that are far more difficult to detect or correct, posing a deeper risk for multi-modal integration (Fig. 2A). To illustrate that design of multiVIB offers a robust solution to alleviates the issue, we set up a conceptual stress test using 10X Genomics Multiome PBMC data[36], a well-established dataset that includes joint RNA and ATAC measurements at the single-cell level, along with granular cell-type annotations. We deliberately blinded models to the true cell correspondences between paired RNA and ATAC measurements in 3 cell types: CD16+ monocytes, CD8+ T cells, and B1 B cells, but used those correspondences as ground truth for validation. For other cell types, models would see paired cells between the RNA and ATAC measurements for learning correspondence between these two modalities. In this setup, we compared cross-modality alignments from multiVIB and other four vertical integration methods: Cobolt [24], SMILE [25], MultiVI [26], and Seurat v5 [27]. We observed that multiVIB showed stronger performance in both modality alignment (modality mixing score) and cross-modality mapping (macro F1) (Fig. 2B). Unlike alternative approaches whose performance degraded as latent dimensionality increased, multiVIB remained robust across a broad range of latent dimensions. Using the local annotation enrichment score (LAES)[12], which measures modality mixing in each cell’s neighborhood, multiVIB also achieved the best local-scale alignment across all three blinded cell types (Fig. 2C).

These results demonstrate that the probabilistic and uncertainty-aware design in multiVIB confers a fundamental advantage in avoiding the hidden failure modes of anchor-based integration, enabling more reliable and biologically coherent alignment even under deliberately adversarial conditions. We next quantitatively evaluate the performance of multiVIB in different integration scenarios against state-of-the-art tools, highlighting its special advantage as a unified framework for mosaic datasets.

### 2.3 multiVIB delivers robust, biologically coherent integration across diverse scenarios

Building a multi-modal cell atlas from collaborative efforts requires integrating mosaic datasets where some modalities are anchored through jointly-profiled cells while others can only be anchored with shared features. It is therefore critical that the integration method performs reliably well in both horizontal (feature-shared) and vertical (joint-profiled) settings. Existing tools often excel in one category or the other but rarely both, forcing researchers to set priorities and selectively sacrifice between batch correction, modality alignment, and biological fidelity when assembling custom hybrid workflows.

multiVIB was designed to avoid this tradeoff: across different integration settings, its unified model backbone should perform equally well to preserve cell-type structure while aligning modalities and removing study-specific variation. To assess whether multiVIB fulfills this promise, we conducted a systematic benchmarking analysis against 9 state-of-the-art approaches using the jointly-profiled 10X Genomics Multiome data, and 4 additional single-modality datasets from independent studies using human PBMC and bone marrow samples[37–40].

We evaluated the integration performance with quantitative metrics in the following aspects[13]: (1) *batch correction* within each modality, measured by batch mixing score; (2) *modality alignment* across modalities, measured by modality mixing score and average silhouette width (ASW) modality; (3) *biological cell-type preservation*, assessed by ASW cell type at both coarse (L1) and granular (L2) label resolutions; and (4) *cross-modality mapping*, measured by overall accuracy and macro F1 score through label transfer task. Details of these metrics are described in Methods. For vertical integration, we compared multiVIB against 4 leading methods: multiVI[26], SMILE[25], Cobolt[24], and Seurat v5 (vertical)[27] (Figure 2D). multiVIB showed superior performance in modality alignment and cross-modality mapping, while all methods achieved comparable scores in cell-type preservation. In addition, multiVIB and Seurat v5 (vertical) outperformed other methods in batch correction for both modalities, underscoring their broader utility. For horizontal integration, we compared multiVIB against 5 state-of-the-art methods: Seurat v3 (horizontal)[18], Scanorama[9], BBKNN[11], Harmony[10], and scVI[7] (Fig. 2E). multiVIB demonstrated consistently strong performance in all four benchmarking categories, as do the leading methods scVI and Seurat v3. Notably, multiVIB excelled simultaneously in modality alignment and batch correction (Supplementary Fig. S1A).

To quantify the balanced performance of multiVIB across tasks, we compared all methods by ranking the average scores across all four benchmarking metrics[13] (Fig. 2D–E). We found that horizontal integration tools (e.g., Harmony, Scanorama, Seurat v3) perform well on cross-studies harmonization but falter on cross-modal alignment; and vertical methods (e.g., Cobolt, SMILE, MultiVI) excel in vertical settings for which they were designed, but struggle to generalize across studies. In contrast, multiVIB maintained top performance across all categories simultaneously. This consistent supreme performance is what enables multiVIB to function as a unified framework for mosaic data integration without manual tuning of integration strategies or custom assembly of workflows.

We further validated whether the unified latent space produced by multiVIB supports biologically meaningful downstream tasks in the multi-modal, multi-study integration, specifically label transfer and cell querying. Label transfer tests the alignment across modalities at the cell type level. Using Granja *et al*. RNA-seq data[38] as the reference and Satpathy *et al*. ATAC-seq[39] as the query, multiVIB transferred cell-type annotations with high concordance to the original biological labels from the independent study[39], (normalized mutual information or NMI = 0.616 and 0.578 for coarse (L1) and detailed (L2) cell-type annotation, respectively, Supplementary Fig. S1B, C). This result demonstrates reliable cross-modal mapping despite substantial differences in data modality and experimental conditions. Cell querying across datasets evaluates whether the unified latent space preserves biologically meaningful neighborhood structure. Again, treating Satpathy *et al*. ATAC-seq data[39] as queries, we identified their 15 nearest neighbors in both 10X[36] and Granja *et al*.[38] RNA-seq datasets. Retrieved neighbors from both studies consistently matched to the query cell types (Supplementary Fig. S1D), demonstrating that multiVIB maintained coherent cell-type structure across datasets and modalities. These results confirmed that multiVIB not only performs well on benchmark metrics but also delivers reliable, biologically grounded alignment that supports real analytical tasks.

### 2.4 Scalable and generalizable mosaic integration of single-cell multi-modal data from mouse primary motor cortex

**Figure 3:**
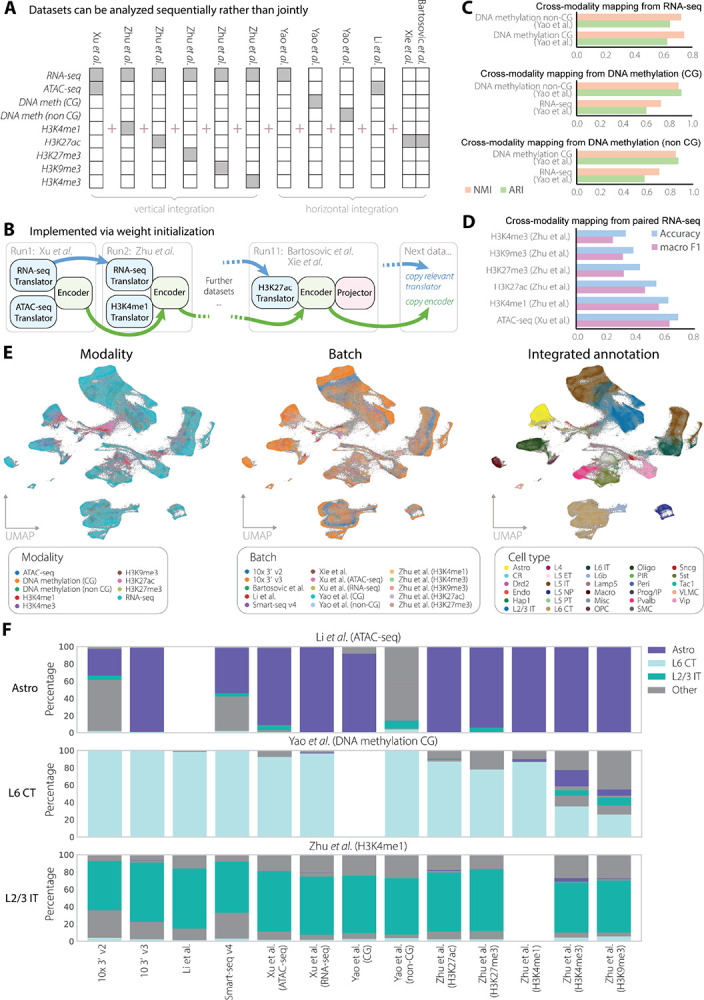
Mosaic integration of mouse motor cortex data over 9 modalities. (A) Overview of MOp data included in this mosaic integration task. Datasets were collected from 6 sources covering 9 modalities. Based on availability of joint measurements, multiVIB was run either in vertical or horizontal mode. (B) Dataset-by-dataset training strategy for integrating MOp data. The encoder always inherits weights from previous training. Translators are updated from previously relevant translators or created from scratch. (C-D) Evaluation of integration with cross-modality mapping, also known as label transfer. (C) Evaluation of horizontal integration. ARI and NMI metrics were used to measure label transfer from one modality to the others. (D) Evaluation of vertical integration. Macro F1 and overall accuracy scores of 6 epigenomic modalities are computed via label transfer from their paired RNA-seq data. (E) Visualization of mouse motor cortex data via UMAP. Cells are colored according to modality sources (left), batch identities (middle), and unified cell type annotations (labels transferred from Yao *et al*. to all cells, right). (F) Cross-study and -modality cell querying. Astrocytes, L6 CT cells, and L2/3 IT cells from 3 different studies (the panels) were queried against all integrated cells to identify the most similar cells in all other data sources (the x-axis labels). Each bar represents the total cell population from each study, color coded by the identified cell types. Aside from the 3 queried cell types, all other cell types are colored gray.

The mouse primary motor cortex (MOp) was the central focus of the first stage of BRAIN Initiative Cell Census Network (BICCN)[41]. As a result, mouse MOp has become one of the brain regions that has been most comprehensively profiled. While the reference atlas has been established using deeply standardized experimental and computational pipelines[42], other research groups have studied the same brain region using additional molecular assays, offering complementary perspectives on neuronal identity and cellular state. Integrating these diverse datasets is key to constructing the next-generation cell atlas that reflects the full molecular complexity of the brain. Here, we assembled a compendium of 9 modalities from 5 additional published sources[22, 23, 43–45], alongside the BICCN reference atlas[42] to create a testbed for evaluating how multiVIB harmonizes diverse omics layers into a coherent cell atlas (Fig. 3A).

In addition to the mosaic data scenario, this integration task presents several common challenges: (1) the dataset composition was heavily skewed toward single-modality scRNA-seq, which is a common imbalance in large single-cell compendium; (2) although jointly-profiled data provided direct RNA–ATAC correspondences as cell anchors, the vast majority of ATAC-seq cells were profiled independently without paired RNA measurements; and (3) no jointly-profiled data linked gene expression and DNA methylation, restricting those modalities to horizontal integration based solely on shared genomic features. Moreover, training all these nine modalities at once is computationally expensive and would prevent more incoming data. To address these practical and conceptual challenges, we implemented multiVIB with its continuous dataset-by-dataset learning strategy: the shared encoder always inherited weights from prior training, while translators were either initialized from previously learned components or trained *de novo* when encountering new modalities (Fig. 3B). This strategy underscored multiVIB’s flexibility to combine vertical and horizontal integration within a unified probabilistic framework, which enables large-scale single-cell atlases to accumulate datasets, including of novel modalities, in an evolving fashion without retraining from scratch.

To validate the biological information in the learned cell embeddings, we evaluated cross-modality mapping of how accurately cell-type annotations could be transferred across datasets and modalities. For horizontal integration tasks, multiVIB achieved high label concordance across 3 modalities (RNA, DNA methylation in CG and non-CG contexts). In particular, both ARI and NMI reached nearly 0.9 in the mutual transfer between CG and non-CG DNA methylation data (Fig. 3C), indicating effective alignment of closely related molecular readouts. For vertical integration tasks, multiVIB achieved label transfer accuracy of 0.63 macro F1 and 0.69 overall accuracy for ATAC-seq data (Fig. 3D). However, the accuracy decreases to 0.23 (macro F1) and 0.32 (overall accuracy) in H3K4me3, potentially reflecting the greater biological distance between transcriptional output and distal epigenetic regulation. UMAP visualization further supported these trends, showing that multiVIB yielded well-mixed cell embeddings across studies and modalities while preserving coherent cell-type structure (Fig. 3E). Together, these results demonstrated that multiVIB reconstructd biologically meaningful relationships across heterogeneous molecular layers and achieved a strong alignment when modalities share regulatory context.

Finally, we show that the unified cell embedding learned by multiVIB empowers biologically meaningful cross-source and cross-modality cell querying. Using representative cell types (astrocytes, L6 CT, and L2/3 IT cells) as queries, we searched for their most similar counterparts across multiple datasets. Query searching consistently retrieved cells with matched identity across many modalities and data sources, demonstrating that the integrated embedding preserved biological correspondence beyond simple alignment (Fig. 3F). However, the agreement decreased in histone modification modalities such as H3K4me3 and H3K9me3, consistent with their lower label transfer performance. This suggested that these layers may capture complementary rather than directly mappable aspects of cell identity (see Discussion).

### 2.5 Disentangling conserved and species-specific molecular programs across primate basal ganglia

The basal ganglia are the primary locus of pathology in motor and cognitive disorders ranging from Parkinson’s to addiction[46–48], making the validation of non-human primate (NHP) models a critical priority for translational neuroscience[5]. While the anatomical organization of basal ganglia is broadly conserved[49], the extent to which molecular programs are shared across human and NHP remains an open question[50]. To establish this translational baseline, the BICAN consortium has generated high-resolution single-cell transcriptomic profiles across human, rhesus macaque, and common marmoset (Johansen *et al*., co-submitted). Using this data, we validated the application of multiVIB in comparative analysis by constructing a unified cross-species landscape and recovering the consensus taxonomy. We further investigated the species-specific embeddings learned by multiVIB to recover distinct transcriptional programs that characterize the human striatum.

**Figure 4:**
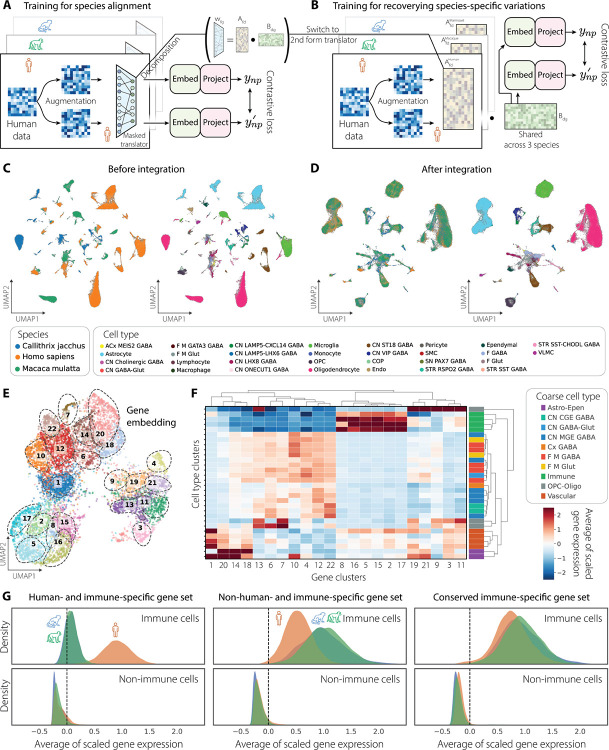
Horizontal integration of basal ganglia data across 3 species. (A) Phase 1 training focuses on species alignment using a masked linear translator to restrict mappings between non-homologous and conserved homologous genes. (B) Phase 2 training recovers additional species-specific variations. The trained translators from phase 1 are decomposed into two lower-rank components, $A_{fd}$ and $B_{dg}$, which are updated without any masking. (C) UMAP visualization of cell embedding before integration. Cells are colored by species and cell type. (D) UMAP visualization of cell embedding after multiVIB integration. (E) Co-embedding of genes from all 3 species. All genes from 3 species are co-embedded through the learned $A_{fd}$ matrices. Then, louvain clustering is applied to group all genes into distinct clusters. (F) Scaled average expression of gene clusters in cell types, illustrating cell-type-specific enrichment. (G) Probability density of averaged scaled gene expression plotted for immune-related gene programs, showing recovery of (1) human-specific, (2) non-human primate-specific, and (3) conserved immune gene programs, obtained from the learned translator matrices after integration. Y-axes are individually scaled, but the area under each curve integrates to 1.

Taking homologous genes as biologically grounded anchors (Johansen *et al*., co-submitted), we implemented multiVIB to integrate transcriptomics data across species under the horizontal principle, utilizing the two-phase training strategy (Fig. 1F). In the first phase, the model focused on cross-species alignment by constraining the mapping within shared features across species only. The mask in the linear translator prevented spurious correlations between genes across species, ensuring that the early goal of model training was cross-species alignment (Fig. 4A). In the second phase, we relaxed this constraint and allowed the model to recover species-specific structure in the data (Fig. 4B).

First, we confirmed that multiVIB effectively aligned human, macaque, and marmoset cells into a unified latent space, grouping transcriptomic profiles by homologous cell types rather than species (Fig. 4C–D). This alignment was consistent with the consensus taxonomy defined by the transcriptomic atlas (Johansen *et al*., co-submitted), with major cell classes and subclass boundaries preserved across species. Next, clustering of the learned gene embedding (Fig. 4E) revealed gene groups with strong cell-type-specific enrichment (Fig. 4F), indicating that multiVIB captured coordinated gene modules that underlie the cellular identity. To further assess multiVIB’s capability of preserving species-specific signals, we examined immune-related gene modules as a representative case (Fig. 4G). Analyzing species-specific matrices learned during training, we recovered three biologically distinct categories of immune gene sets: human-specific, NHP–specific, and conserved across all three species (details in Methods). Successful retrieval of coherent, species-resolved gene programs demonstrated that multiVIB not only aligned shared cell identities but also preserved evolutionarily meaningful divergence.

## 3 Discussion

A central task of single-cell biology is to construct multi-modal atlases that map a full diversity of cellular states at different molecular resolutions. The BRAIN Initiative has been advancing this vision with rapid generation of expansive transcriptomic and epigenomic datasets[51]. With reference atlases built by individual centers with standard pipelines, and datasets only analyzed within the designed studies, previous computational tools were developed mostly to address only narrow scenarios. multiVIB, as a unified computational framework capable of integrating increasingly heterogeneous datasets while preserving biological fidelity, serves as the critical missing piece. By combining a minimalist model backbone with a probabilistic contrastive learning strategy, multiVIB adapts to diverse integration scenarios and mitigates artifacts such as spurious alignment that often arise in multi-modal data integration. As a result, it enables continuous evolution of reference atlases with growing data (Fig. 3), and connects multiple species to empower comparative analysis (Fig. 4).

### 3.1 Principles for atlas-scale single-cell integration

Our experience applying multiVIB to more than one million cells across BICCN and BICAN datasets revealed several general principles that extend beyond any individual method. These principles reflect recurring patterns in multi-modal or multi-species atlas construction and help explain why naive or purely technical integration strategies often fail to preserve true biological variation.

First, data representativeness is critical. Jointly-profiled multi-omics datasets serve as powerful anchors for vertical integration, yet they remain sparse relative to the scale of single-modality datasets and often undersample rare or transitional cell populations. Over-reliance on these incomplete anchors can propagate their sampling biases, resulting in spurious alignments or the collapse of biologically meaningful states.

Second, integration must respect biological interpretability. Attempts to coerce heterogeneous assays into superficially shared feature spaces (e.g., summarizing chromatin conformation or accessibility peak counts as “gene activity” proxies) can distort underlying regulatory logic. Our analyses across nine modalities in the motor cortex showed that such engineered correspondences frequently obscure modality-specific information rather than facilitating true integration.

Third, multi-modal integration requires semantic awareness. Modalities that appear structurally similar may encode opposing regulatory states. For example, chromatin accessibility and DNA methylation can reference the same genomic regions yet convey inverted functional meanings. Treating such modalities as interchangeable features may misinterpret the very biological signals that are needed to distinguish conserved programs from those that are species- or lineage-specific.

Taken together, these insights highlight that effective multi-modal integration is not merely a statistical harmonization task but fundamentally a problem of biological inference. Preserving the diversity of regulatory mechanisms across assays, species, and modalities is essential for reconstructing cellular identity at atlas scale. Principles above clarify a broader role of multiVIB within the BRAIN Initiative. By explicitly modeling uncertainty, multiVIB avoids both insufficient alignment and over-correction, while preserving the semantic structure of each modality. In doing so, it provides an integration framework that scales with the rapidly expanding scope of BICAN. The challenges encountered in integrating BICCN (multi-modal motor cortex) and BICAN (cross-species basal ganglia) datasets will only intensify as additional modalities, developmental stages, and disease contexts are incorporated. In this context, these principles would also offer a generalizable computational foundation for constructing the next generation of comprehensive, multi-modal brain cell atlases.

### 3.2 Addressing spurious alignment in single-cell data integration

Spurious alignment remains a persistent challenge in single-cell data integration and poses a major risk when constructing reference atlases from federated data sources. It occurs when multiple mathematically plausible alignments exist among datasets, leading models to converge on solutions that primarily minimize technical variation rather than capturing true biological correspondence. This issue can arise in both horizontal and vertical integration. In horizontal integration, spurious alignment often results from poorly defined or improperly engineered cross-modal shared features. In vertical integration, the risk increases when paired cells in jointly-profiled datasets fail to represent the full spectrum of cellular diversity. multiVIB mitigates spurious alignment in both scenarios through its modular translator architecture and stochastic cell embeddings: for horizontal integration, through the two-phase design of the modality-specific translators; and under vertical integration, through probabilistic cell embeddings, where contrastive loss directly enhances cross-modality similarity between paired cells. Therefore multiVIB was designed specifically to strengthen biological correspondence across datasets instead of relying on reconstruction-based objectives to approximate a unified latent space.

### 3.3 Supporting ontology-level cell type harmonization

A major objective of many consortium projects, including the BRAIN Initiative, is to establish unified and interoperable cell-type taxonomies across datasets, modalities, and species[1]. Despite ongoing community efforts to standardize nomenclature and ontology, practical approach for data harmonization remains challenging, as each study often defines cell populations at different resolutions or through distinct molecular modalities. This challenge has motivated the development of automated tools such as CellHint[52], which performed cross-dataset label harmonization by identifying equivalent cell populations in the shared cell embedding. Our results showed that the unified latent space learned by multiVIB provides a strong computational foundation for ontology-level harmonization. When CellHint was applied to multiVIB-derived embeddings, it achieved high cross-study concordance across transcriptomic, chromatin accessibility, DNA methylation, and histone modification datasets, indicating that the learned representations captured biologically coherent cell relationships that generalized effectively to external harmonization frameworks (Supplementary Fig. S2). This capability positions multiVIB not only as a method for data integration but also as an enabling infrastructure for the BRAIN Initiative’s broader goal of building interoperable, cross-consortium cell atlases grounded in a unified cell ontology.

### 3.4 Beyond unification: embracing modality-specific biological variation

While learning a unified cell embedding across studies, species, and modalities is an enticing goal for building a comprehensive cell atlas, our results suggest this may not be biologically attainable in certain cases. For example, we noticed some major cell types were poorly separated using histone modification features such as H3K9me3 and H3K4me3. Although this could reflect technical limitations, such as insufficient measurement or experimental sensitivity, it is also possible that some epigenetic marks simply cannot encode cell identity at the same level of specificity as transcriptional or chromatin accessibility signals. Consistent with this interpretation, we observed that current computational methods, including multiVIB, performed worse in cross-modality label transfer compared to within-modality label transfer, which could reflect fundamental biological divergence across omics layers besides technical limitations. These findings suggest that future efforts on the development of computational methods should prioritize extracting modality-specific biology instead of imposing a universal embedding for all modalities. Methods that can better capture the complementary roles of distinct molecular modalities in defining cellular state and function may result in a more faithful and multidimensional representation of cell identities.

## 4 Methods

### 4.1 Probabilistic contrastive learning

We approximate the latent representation using an encoder network that produces a posterior distribution in the latent space, and we train the model using a contrastive loss. These elements form the core of probabilistic contrastive learning in our study.

The conceptual framework for the loss function in multiVIB has roots in the information bottleneck (IB) study [53]. The objective of IB is to maximize the mutual information contained in latent representation z from input data x, while restricting how much information about the identity of each data element is allowed in this representation. Under the information bottleneck principle, a variational form of IB (VIB) [32] can be set up as:

(1) $${ℒ}_{\text{multiVIB}}={ℒ}_{\text{DC}}+\beta D_{\text{KL}}\left[q_{\phi }\left(z_{nk}|x_{ng}\right)\|p\left(z_{nk}\right)\right]$$

where ${ℒ}_{\text{DC}}$ is our task-specific contrastive loss and $q_{\phi }\left(z_{nk}|x_{ng}\right)$ is the variational posterior distribution for the latent variables conditioned on the data, implemented as an encoder network, and $p\left(z_{nk}\right)$ is the prior over latent representation. $D_{\text{KL}}[\cdot \|\cdot ]$ represents the KL divergence. The hyperparameter β sets the bottleneck strength, which controls the amount of information retained in $z_{nk}$. In the Eq. (1), the KL divergence term ensures this distribution does not stray too far from the prior. In this study, we set β as 0.05 for all data integration.

Contrastive learning has been successfully applied to learn representations of images in numerous studies[54, 55]. This was soon copied to other data types, including single-cell omics data[25, 56–58]. The key component of contrastive learning is to use data augmentation to create positive pairs that represent different “views” of the same input data. These positive pairs, along with negative examples, are combined to compute a contrastive loss, which maximizes embedding similarity between positive pairs while maximizing differences among negative examples. We use the “decoupled contrastive learning” (DC) loss from Yeh *et al*. [59].

During horizontal integration, we apply this contrastive loss to the projector outputs $y_{np}$, as written above (Eq. 4). The (v) superscript refers to two augmented views of the same data point. The loss terms associated with cell n would be written:

(2) $$U_{n,v}=\sum_{l\in \{1,2\},j\in \{\text{range}(1,N)|j\neq n\}}\text{exp}\left(\text{cos}\left(y_{np}^{\left(v\right)},y_{jp}^{\left(l\right)}\right)/\tau \right),$$

(3) $${ℒ}_{\text{DC},n}^{\left(v\right)}=-\text{cos}\left(y_{np}^{\left(1\right)},y_{np}^{\left(2\right)}\right)/\tau +\text{log}\;U_{n,v},$$

(4) $${ℒ}_{\text{DC}}^{\text{horizontal}}=\sum_{v\in \{1,2\},n\in \text{range}(1,N)}{ℒ}_{\text{DC},n}^{\left(v\right)}.$$

$U_{n,v}$ is a latent space distance metric summed over all pairs of negative examples for the view v of cell n. The sum is composed of 2(N-1) terms, since each example has two augmented views, where N is the number of cells in a minibatch.

During vertical integration, we use the latent space z to compute the loss instead. The full loss for vertical data integration (again for the case of two modalities A and B) is given in Eq. 7. In the vertical case, the (v) superscript refers to measured data from different modalities rather than data views created by augmentation. For example if we are choosing two modalities (A and B) as our positive examples (different views of the same data), the loss terms associated with cell n would be written

(5) $$U_{n,v}=\sum_{l\in \{\text{A},\text{B}\},j\in \{\text{range}(1,N)|j\neq n\}}\text{exp}\left(\text{cos}\left(z_{nk}^{\left(v\right)},z_{jk}^{\left(l\right)}\right)/\tau \right)$$

(6) $${ℒ}_{\text{DC},n}^{\left(v\right)}=-\text{cos}\left(z_{nk}^{\text{A}},z_{nk}^{\text{B}}\right)/\tau +\text{log}\;U_{n,v}$$

(7) $${ℒ}_{\text{DC}}^{\text{vertical}}=\sum_{v\in \{\text{A},\text{B}\},n\in \text{range}(1,N)}{ℒ}_{\text{DC},n}^{\left(v\right)}$$

The “probabilistic” nature of multiVIB comes from the fact that the variational encoder defines a distribution over the latent space, rather than a single deterministic embedding. A probabilistic embedding adds quantifiable uncertainty to the cell embedding[33, 34]. We argue this kind of uncertainty is critical for capturing the (possibly limited) information coming from one data modality or another. The relevant thought experiment would be inventing a new data modality which introduces a new variant of of T cell. This new variant of T cell is not seen in model training with other modalities. Embedding cells as probability distributions is beneficial for dealing with cases like this thought experiment. When the model cannot directly learn the embedding for cell states that are missing in a reference dataset, embeddings of these cell states across modalities can be inferred through their closest cell states. In this thought experiment, cell embedding of this new variant of T cell is inferred through other variants of T cells that are seen in other modalities. Meanwhile, such inference would also limit spurious alignment across modalities, from the standpoint of data integration.

### 4.2 The backbone of multiVIB

The model backbone of multiVIB consists of three parts: (1) a modality-specific linear translator, (2) a shared encoder, and (3) a shared projector.

#### Notation—

Let $x_{nf}^{\left(m\right)}$ denote the observed feature vector for cell n in modality m, where f indexes modality-specific features. Let g index genes in the shared gene-centric feature space, k the dimensionality of the shared latent cell embedding, and p>k the dimensionality of the final projection space. Covariates such as batch, species, or modality are denoted $C_{n}$. The backbone of multiVIB consists of three mappings:

(8) $$x_{nf}^{\left(m\right)}\overset{T^{\left(m\right)}\;}{\to }h_{ng}\overset{{\text{NN}}_{\text{encoder}}\;}{\to }z_{nk}\overset{P\;}{\to }y_{np}.$$

Each modality-specific translator $T^{\left(m\right)}$ is implemented as a linear map with weights $W^{\left(m\right)}\in R^{f\times g}$ and bias $b_{g}^{\left(m\right)}\in R^{g}$.

#### Modality-specific translator—

Each modality m is equipped with a linear translator mapping modality-specific features into the shared gene-centric space:

(9) $$h_{ng}=T^{\left(m\right)}\left(x_{n\cdot }^{\left(m\right)}\right)=\sum_{f}w_{fg}^{\left(m\right)}x_{nf}^{\left(m\right)}+b_{g}^{\left(m\right)}.$$

The gene-centered translated space $h_{ng}$ leverages the abundance of scRNA-seq datasets while providing an interpretable coordinate system. The linear structure of $T^{\left(m\right)}$ makes $W_{fg}^{\left(m\right)}$ and $b_{g}^{\left(m\right)}$ directly interpretable for uncovering cross-modal feature relationships and regulatory interactions.

We implemented two different forms of translators for distinct purposes. The first form of the modality-specific translator is implemented as a fully-connected dense layer. The original input $x_{nf}^{\left(m\right)}$ is multiplied by $w_{fg}^{\left(m\right)}$ and summed with a bias term $b_{g}^{\left(m\right)}$, giving the translated output $h_{ng}^{\left(m\right)}$, where f stands for the original feature space and g is the shared feature dimension. However, this dense layer could learn implausible correlations between features f and g, which we believe would be a major source of spurious alignment. Therefore we add a binary mask matrix, $m_{fg}\in \{0,1\}$, informed by a biological prior, to force the model to explore only “biologically-plausible” correlations between the original feature space f and shared feature space g (Eq. (10)).

Such a biological prior can come in the form of two features that are physically close in the genome. For example, opening of a promoter region in ATAC-seq data is more likely to influence transcription of the proximal gene than a distant gene. $w_{fg}^{\left(m\right)}$ is sparse, due to the sparsity imposed by the mask. Meanwhile, imposing the mask also prevents the translator to recovery dataset-specific variations. Therefore, we introduce the second form of the translator through lower-rank matrix decomposition (Eq. (11)). The masked weight matrix $w_{fg}m_{fg}$ is decomposed into $A_{fd}^{\left(m\right)}$ and $B_{dg}^{\left(m\right)}$ using singular value decomposition (Eq. (12)). d is a compressed dimension (128 in multiVIB), making this scheme equivalent to allowing only low-rank updates to the weight matrix. Without mask, the second form of the modality-specific translator gives the model more flexibility to explore feature correlations between the original and shared feature space. In the training detail, we will further explain how we interchange these two forms to serve integration purpose.


(10) $$h_{ng}=x_{nf}\left(w_{fg}m_{fg}\right)+b_{g}$$

(11) $$h_{ng}=x_{nf}\left(A_{fd}B_{dg}\right)+b_{g}$$

(12) $$\left(w_{fg}m_{fg}\right)\;\approx A_{fd}B_{dg}$$

#### Shared probabilistic encoder—

The shared encoder, denoted ${\text{NN}}_{\text{encoder}}$, produces the parameters of a Gaussian distribution rather than a single deterministic embedding. Specifically, it outputs a cell-specific mean and log-scale of a Gaussian posterior distribution:

(13) $$z_{nk;\mu }\left(h_{n\cdot }\right),\;z_{nk;\sigma }\left(h_{n\cdot }\right)={\text{NN}}_{\text{encoder}}\left(h_{n\cdot }\right),$$

where σ denotes the (diagonal) standard deviation. A stochastic latent representation $z_{nk}$ is then obtained via the reparameterization trick. This probabilistic formulation, along with a standard normal prior imposed on $z_{nk}$ imposes an information bottleneck analogous to VAEs, stabilizes training, and regularizes the latent biological state. The resulting $z_{nk}$ serves as a modality-agnostic embedding across datasets.

$z_{nk}$ is a K-dimensional latent variable representing the “unified” cell embedding, with a standard normal prior (Eq. 14). The encoder ${\text{NN}}_{\text{encoder}}$ is implemented as a multi-layer perceptron. Here, we use a fully-connected dense neural network with 3 hidden layers. The encoder generates sufficient statistics for the posterior distribution $q_{\phi }\left(z_{nk}|x_{ng^{\prime }}\right)$, namely $z_{nk;\mu }\left[x_{ng^{\prime }}\right]$ and $z_{nk;\sigma }\left[x_{ng^{\prime }}\right]$ (Eq. 15). The posterior $q_{\phi }\left(z_{nk}|x_{ng^{\prime }}\right)$ follows a normal distribution (Eq. 16). Here ϕ represents the bundle of parameters in the translator and encoder. We update these model parameters using variational inference[32, 60].


(14) $$z_{nk}\sim 𝒩(0,1)$$

(15) $$z_{nk;\mu }\left[x_{ng^{\prime }}\right],\;z_{nk;\sigma }\left[x_{ng^{\prime }}\right]={\text{NN}}_{\text{encoder}}\left(x_{ng^{\prime }}\right)$$

(16) $$z_{nk}|x_{ng^{\prime }}\sim 𝒩\left(z_{nk;\mu }\left[x_{ng^{\prime }}\right],z_{nk;\sigma }\left[x_{ng^{\prime }}\right]\right)$$

#### Shared projector—

To address batch, species and modality differences during horizontal integration, $z_{nk}$ is concatenated with one-hot encoded batch, species, or modality variables ${\vec{C}}_{n}$, and the shared projector then transforms the concatenated variables into a slightly higher-dimensional space $y_{np}$. In this study, the shared projector is implemented as a single fully-connected dense layer, without any batch normalization or activation function.

(17) $$y_{np}=P\left(\left[z_{nk},C_{n}\right]\right),$$

where p>k. By explicitly conditioning on covariates, the projector absorbs covariates-related variation, enabling $z_{nk}$ to capture primarily biologically meaningful structure.

### 4.3 Horizontal integration with multiVIB

In phase 1 of training, the modality-specific translator requires one-to-one or many-to-one correspondences between original and translated features in the form of a mask. For example, a translator for scATAC-seq data (translating to gene space) can use a mask based on the criterion that regions within +/−2000bp of a gene are likely to contribute to transcription of that gene, while distal regions have less impact on its transcription. Masked out weights in the translator will be inactive (zero) during phase 1 training. This enables exploration of biologically-informed correlations while preventing the model from learning spurious associations. After initial phase 1 training (100 epochs in this study), we switch to phase 2, lifting the mask constraint from the translator and allowing all weights to be updated. We keep training model for extra epochs (200 epochs in this study), and the second form of the translator should enable the model to capture a broader range of correlations between the original features and the shared space.

### 4.4 Vertical integration with multiVIB

When cells are jointly profiled to obtain multi-omics data, so that an individual cell has two data modalities measured, we first fit a linear regression model with sklearn.linear_model.LinearRegression, which estimates regression coefficients that map features from one modality to the other by minimizing the sum of the squared differences between predicted and observed values. This step discovers correlations between original and translated features, and is used to initialize the linear modality-specific translator. In vertical integration, we primarily skip phase 1 training, acting as though the linear regression results are the output of phase 1, and we directly use the second form as the translator, where the weight matrix is factorized into $A_{fd}$ and $B_{dg}$. This reduces the number of trainable parameters. However, users have the option to train using phase 1 training when needed.

### 4.5 Input data pre-processing

For scRNA-seq data, we took common pre-processing steps by (1) normalizing the total counts to 10,000 scale, (2) log1p-transforming, and (3) standardizing gene features by removing the mean and scaling to unit variance. For scATAC-seq data, we perform a TF-IDF transformation on the accessibility matrix[61]. For a peak p in cell n, the transformed TF-IDF peak matrix $t_{np}$ is calculated as:

(18) $$t_{np}=\text{TFIDF}(p,n)=\text{TF}(p,n)*\text{IDF}(p)$$

(19) $$\text{TF}(p,n)=\frac{x_{np}}{\sum_{p^{\prime }}x_{np^{\prime }}}$$

(20) $$\text{IDF}(p)=\text{log}\left(\frac{1+N}{1+N_{p}}+1\right)$$

(21) $$N_{p}=\sum_{n}1\left[x_{np}>0\right]$$

where $x_{np}$ is the original peak count data, N is the total number of cells, and $N_{p}$ is the number of cells that have peak p. We further standardize the transformed accessibility matrix $t_{np}$ by removing the mean and scaling to unit variance.

For histone modification data, we bin peak features into 10,000bp windows. We log1p-transform the binned data and standardize the histone modification matrix by removing the mean and scaling to unit variance. Since, all input datasets are standardized by removing the mean and scaling to unit variance, we adapt a universal augmentation for all modalities via adding random gaussian noise with mean as 0 and variance as 1 to individual features and cells. We also implement 0.25 feature dropout in the shared encoder to randomly shutdown certain paths from translated features $h_{ng}$ to latent representation $z_{nk}$, adding additional augmentations to the input data.

We use scRNA-seq datasets to call out 2000 highly variable genes in each integration task, and these 2000 HVG would serve as the shared gene-centric features. All other modalities will first be translated into the shared 2000 gene feature space through modality specific translator $T^{\left(m\right)}$.

### 4.6 Evaluation of mouse motor cortex data

For mouse primary motor cortex data, jointly-profiled multi-omics data provides multiple data modality measurements per cell. For the purposes of evaluation, we hold out 20% of cells as a test set and use the remaining 80% of cells for training using vertical integration.

### 4.7 Identification of species-specific gene sets

In phase 2 training, the modality translator consists of 2 matrices $A_{fd}$ and $B_{dg}$. In cross-species integration (Fig. 4B), we obtain 3 species-specific $A_{fd}$ matrices and 1 shared $B_{dg}$. $A_{fd}^{\text{Human}},A_{fd}^{\text{Marmoset}}$, and $A_{fd}^{\text{Macaque}}$ capture species-level regulatory patterns, while $B_{dg}$ represents a common mapping to the conserved genes across species. We first clustered shared homologous genes into groups using the matrix $B_{dg}$ and categorized them based on their expression patterns in cell types. Then, we reconstructed a mapping from species-specific genes to shared genes by multiplying $A_{fd}$ and $B_{dg}$ to recover $w_{fg}$ separately for each species. For a given cell type, shared genes that are categorized as being enriched in that cell type were selected. Next, we calculated a z-score of all individual weights of species-specific genes that are linked to shared genes of interest using $w_{fg}$ in each species, and we defined species-specific genes that have z-scores larger than 2.3263 (equivalent to p-value of 0.01) with at least 5 shared genes as significant in that species. This procedure returns species-specific gene sets that are highly correlated with at least 5 shared homologous genes of interest, which could be further interpreted to have a potential regulatory roles in that species, given a cell type of interest.

### 4.8 Evaluation metrics

#### 4.8.1 Batch and modality mixing score

We adapted an entropy-based calculation to measure mixing of batch or modality from Xiong *et al*.[62]. Batch or modality mixing score measures a regional mixing of cells from different batches or modalities, with a high score suggesting cells from different sources are well mixed. This score is computed as

choosecelln

$$p_{i}^{\left(n\right)}=\text{fraction}\;\text{of}\;\text{nearest}\;100\;\text{neighbors}\;\text{from}\;\text{modality}\;i$$

(22) $$E^{\left(n\right)}=\sum_{i}p_{i}^{\left(n\right)}\;\text{log}\left(p_{i}^{\left(n\right)}\right)$$

(23) $$E=\frac{1}{100}\sum_{n=1}^{100}E^{\left(n\right)}$$

In our calculation, a given region is represented by a random sample of 100 cells from each batch or modality. $p_{i}^{\left(n\right)}$ is the fraction of cells from batch or modality i in the 100 nearest neighbors of cell n. This process is repeated 100 times to get an averaged batch or modality mixing score E.

#### 4.8.2 ASW

We adapted averaged silhouette width score (ASW) from Luecken et al. to measure the relationship between the within-cluster distances of a cell and the between-cluster distances of that cell to the closest cluster[13].

To evaluate data integration outputs, we calculated ASW scores with (1) cell type labels (“ASW cell type”) and (2) combined cell type labels and modality labels to measure modality mixing (“ASW modality”).

(24) $${\text{ASW}}_{\text{cell}\;\text{type}}=\left({\text{ASW}}_{c}+1\right)/2$$

(25) $${\text{ASW}}_{\text{modality}}=\frac{1}{C}\sum \left(1-\left|{\text{ASW}}_{\text{m}}\left(j\right)\right|\right)$$

where ${\text{ASW}}_{c}$ is a classical definition of silhouette width score computed using sklearn.metrics.silhouette_score. c denotes the set of cell type labels. For each cell type $j,{\text{ASW}}_{\text{m}}(j)$ is calculation of classical silhouette width score computed using modality label. Then, the final ${\text{ASW}}_{\text{modality}}$ is the average score of $1-\left|{\text{ASW}}_{\text{m}}(j)\right|$ across all cell types C.

#### 4.8.3 Label transfer

Label transfer evaluates cross-modality mapping by assessing how accurately cell-type annotations from one modality can be predicted in another. We quantified label transfer performance using overall accuracy and macro F1 score. These two metrics are used when the source data and target data has the same annotation resolution. Accuracy presents an overall prediction accuracy, regardless of cell types. On the other hand, macro F1 shows an average prediction across cell types. Thus, a low macro F1 score but high overall accuracy would indicate inaccurate prediction for minor cell types.

#### 4.8.4 Local annotation enrichment score

To evaluate modality mixing at a local neighborhood scale, we designed the metric, local annotation enrichment score (LAES)[12]. For each cell type, we calculate the k-nearest neighbors of individual cells in the unified latent space $z_{nk}$. The per-cell ${\text{LAES}}_{n}$ and mean LAES for the cell-type population are calculated as:

(26) $${\text{LAES}}_{n}=\left|\left(\frac{N_{\text{A}}^{\left(k\right)}}{N_{\text{A}}}-\frac{N_{\text{non-A}}^{\left(k\right)}}{N_{\text{non-A}}}\right)*\left(\frac{N}{k}\right)\right|$$

(27) $$\text{LAES}=\frac{1}{n}\sum_{n}{\text{LAES}}_{n}$$

where n is the index of a cell in modality $\text{A},N_{\text{A}}^{\left(k\right)}$ is the number of cells in modality A among the k-nearest neighbors of cell $n,N_{\text{non-A}}^{\left(k\right)}$ is the number of cells in all other modalities among the k-nearest neighbors of cell n,k is the number of nearest neighbors used in the computation, and N is the total number of cells of a certain cell type combining all modalities.

LAES estimates the local discrepancy between density of points from two or more sources: in this case, cells from different modalities. LAES close to 0 indicates that samples from these sources may come from the same distribution, which suggests good modality mixing in the latent space $z_{nk}$.

#### 4.8.5 NMI

We measure the normalized mutual information (NMI) with the sklearn implementation: sklearn.metrics.normalized_mutual_info_score. NMI compares the overlap of two clusterings. We used NMI in this study to compare the author-reported cell-type labels with label transfer predictions from another source of dataset. Due to different annotation resolutions of datasets from different studies, NMI scores of 0 or 1 correspond to uncorrelated annotation or a perfect match of annotation resolution, respectively.

#### 4.8.6 ARI

The adjusted rand index (ARI) is implemented with sklearn.metrics.adjusted_rand_score. ARI also compares the overlap of two clusterings, and we also included ARI in this study to compare the author-reported cell-type labels with label transfer predictions from another source of dataset. ARI is more sensitive to resolutions of two labels, compared to NMI. If two labels from two different sources have distinct annotation resolutions, the comparison usually returns low ARI score close to 0. Only if resolutions of two labels are very close, the calculation will yield ARI score close to 1.

#### 4.8.7 Average score for method ranking

To report a method ranking in benchmarking, we first ranked methods in individual evaluation metrics. Over 4 evaluation categories, we averaged the ranking index of each method in each evaluation category. We then calculated the final ranking index R of method i as below:

(28) $${\text{R}}_{i}=R_{\text{BCS}}*0.2+R_{\text{MAS}}*0.2+R_{\text{CPS}}*0.3+R_{\text{CMS}}*0.3$$

where BCS stands for batch correction score, MAS for modality alignment score, CPS for cell-type preservation score, and CMS for cross-modality mapping score.

Our averaged ranking gives higher weights on biological information-related metrics (preservation of biological variation and cross-modality mapping).

## Supplementary Material

1

## Data Availability

10X Multiome PBMC dataset was accessed via the NeurIPS 2021 Datasets and Benchmarks track. Other human PBMC and bone marrow datasets used in this study could be found through the original studies, with Gene Expression Omnibus (GEO) accession numbers GSE139369, GSE129785, and GSE216005, respectively. Preprocessed cross-species basal ganglia datasets can also be found at https://alleninstitute.github.io/HMBA_BasalGanglia_Consensus_Taxonomy/. Previously collected mouse primary motor cortex datasets of RNA-seq, DNA methylation, and ATAC-seq under BICCN initiative can be found under NeMO https://nemoarchive.org/. Preprocessed versions of these datasets are also available at https://cellxgene.cziscience.com/datasets. Joint profiling mouse primary motor cortex data of gene expression and chromatin accessibility through ISSAAC-seq was deposited at ArrayExpress under the accession number E-MTAB-11264. Joint profiling mouse primary motor cortex data with histone modifications through Pair-tag was deposited at GEO under accession number GSE152020.

## Code Availability

multiVIB code is available at https://github.com/broadinstitute/multiVIB. Tutorials using multiVIB for data integration are also provided in the multiVIB GitHub repository.
