# Supplementary material for: multiVIB: A unified probabilistic contrastive learning framework for atlas-scale integration of single-cell multi-omics data
Source: bioRxiv. 2025 Dec 1:2025.11.29.691308. Preprint. [Version 1] doi: 10.1101/2025.11.29.691308 (PMC12694596; doi:10.1101/2025.11.29.691308)
Supplement: 1 [file NIHPP2025.11.29.691308V1-supplement-1.pdf]

bioRxiv preprint doi: https://doi.org/10.1101/2025.11.29.691308; this version posted December 1, 2025. The copyright holder for this preprint (which was not certified by peer review) is the author/funder, who has granted bioRxiv a license to display the preprint in perpetuity. It is made available under aCC-BY 4.0 International license.

S Supplemental Figures

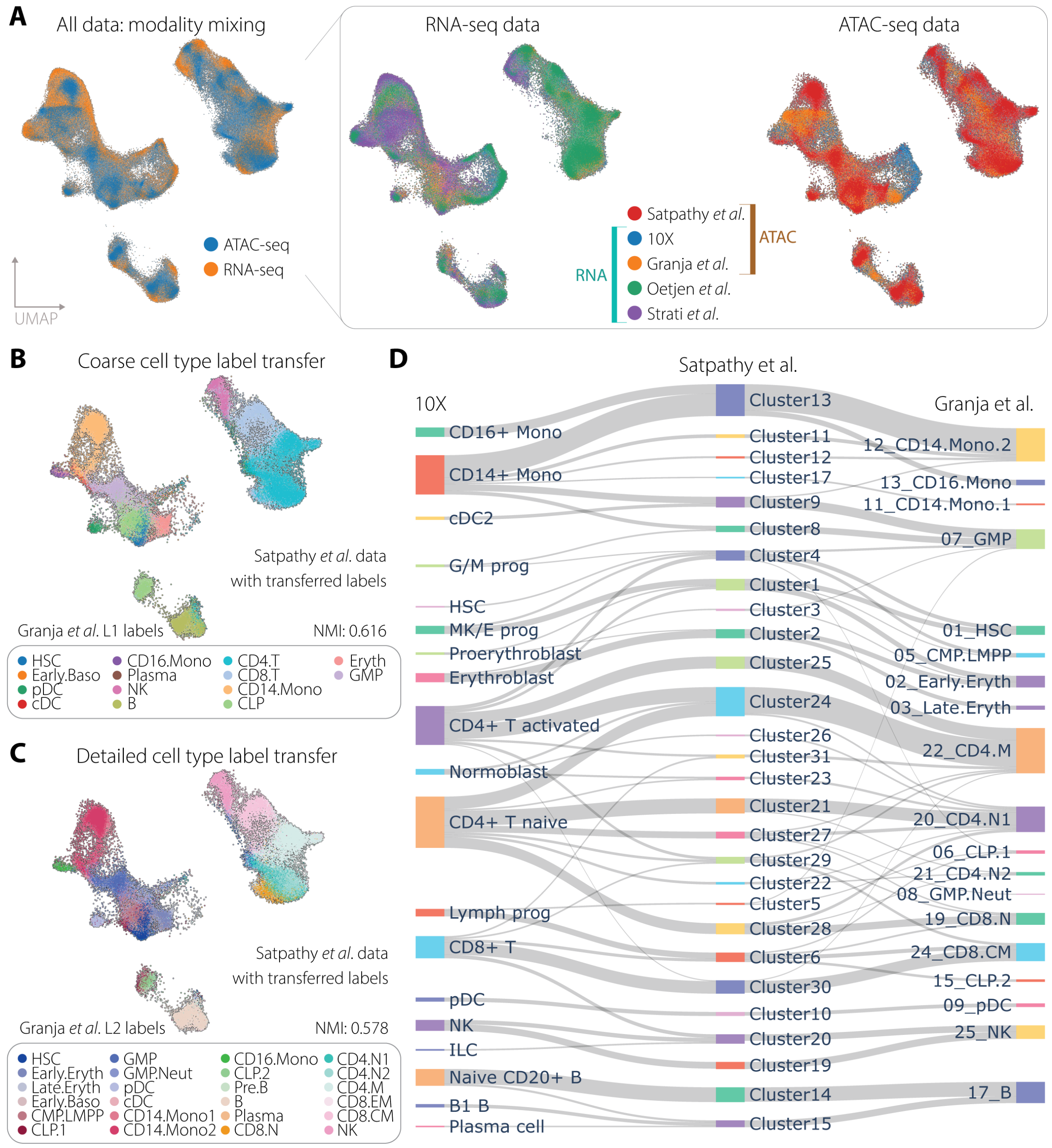

Supplementary Figure 1: multiVIB enables label transfer and cell querying. (A) UMAP visualization of multi-study human PBMC data integration via multiVIB (vertical). Cells are colored according to modality source (left), batch ID of gene expression modality (middle), and batch ID of chromatin accessibility modality (right). (B, C) Label transfer from Granja *et al.* scRNA-seq data to Satpathy *et al.* scATAC-seq data. Transferred labels are evaluated with author-reported clustering label using NMI. (B) Coarse cell type labels (L1) were transferred. (C) Detailed, granular cell type labels (L2) were transferred. (D) Mapping cells of Satpathy *et al.* scATAC-seq data to 10X and Granja *et al.* scRNA-seq data. The top 15 similar cells in 10X and Granja *et al.* scRNA-seq data for each cell in Satpathy *et al.* scATAC-seq data were retrieved using the multiVIB latent space. Majority voting is used to identify the most similar cell type for each cell of Satpathy *et al.* scATAC-seq data. The gray connectors link cells from the query Satpathy *et al.* scATAC-seq data to the other two reference datasets.

bioRxiv preprint doi: https://doi.org/10.1101/2025.11.29.691308; this version posted December 1, 2025. The copyright holder for this preprint (which was not certified by peer review) is the author/funder, who has granted bioRxiv a license to display the preprint in perpetuity. It is made available under aCC-BY 4.0 International license.

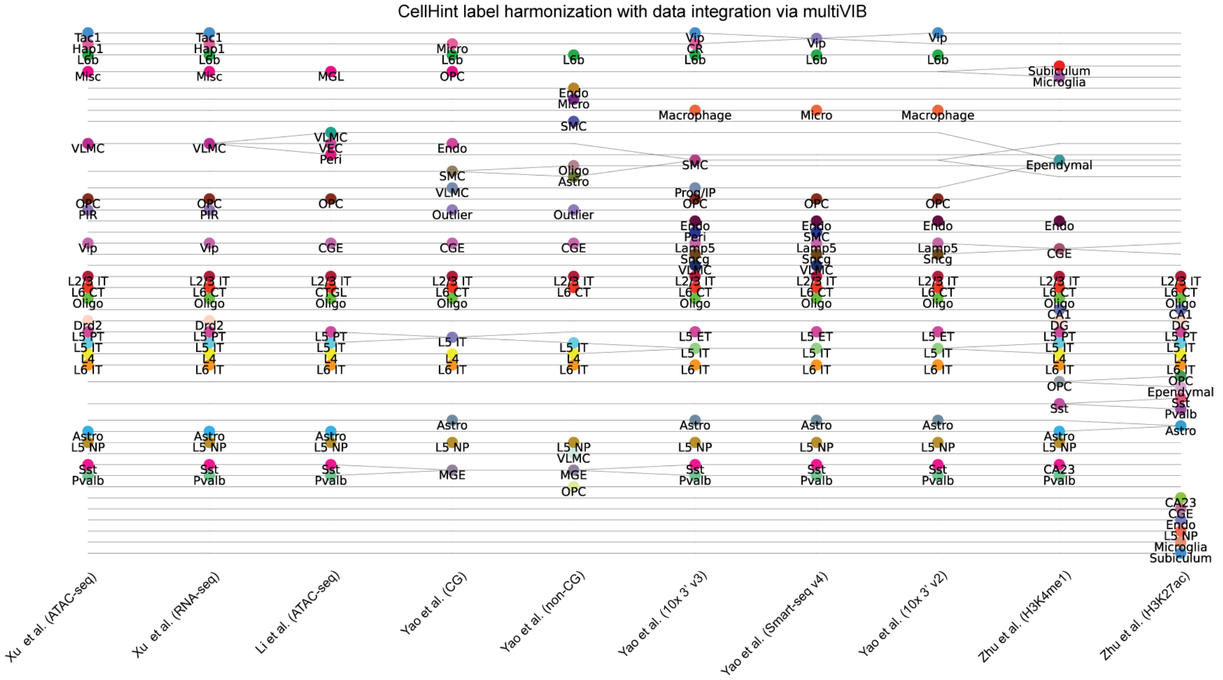

Supplementary Figure 2: CellHint annotation harmonization of mouse primary cortex data with multiVIB cell embedding. Mapping cell types across modalities is computed using the tool CellHint. CellHint takes cell embeddings and cell type labels to harmonize cell type labels across datasets. Here, we applied CellHint to map the consensus cell type across modalities. For cell embeddings, we used the unified cell embeddings learned with multiVIB. Using multiVIB cell embedding CellHint accurately mapped cell types that have the same identity across modalities, with exception that some cell types of H3K27ac data modality cannot be accurately matched with other modalities.
